# Salvaging Chronic Nonunion of Femoral Neck and Infected Nonunion of Ipsilateral Femoral Shaft Fracture Using Intramedullary Antibiotic Cement Spacer and External Fixator Alone

**DOI:** 10.7759/cureus.12665

**Published:** 2021-01-12

**Authors:** Arvind Kumar, Rizwan Khan, Javed Jameel, Sandeep Kumar

**Affiliations:** 1 Orthopaedics, Hamdard Institute of Medical Sciences and Research, New Delhi, IND

**Keywords:** infection, intramedullary cement spacer, ipsilateral femoral neck shaft, neck femur, non-union, rail fixator, shaft femur

## Abstract

Concomitant ipsilateral fractures of femoral neck and shaft are rare injuries and pose challenging management. Infected non-unions of such fractures can further complicate the management options and have not been discussed in the literature. We present a case of an eight-month-old atrophic non-union of ipsilateral femoral shaft and femoral neck with evidence of intramedullary infection that was managed using a cost-effective, low strain rail fixator assembly and an intramedullary antibiotic cement spacer. Both fracture non-unions were salvaged without the need for any additional procedure. The patient returned to his regular activities within a year follow-up period. There was no clinical evidence of infection during the last follow-up at 16 months, and inflammatory markers were within normal limits. The current case study suggests that while aggressive debridement and intramedullary antibiotic cement spacer can control the intramedullary infection, and simultaneous union of even atrophic nonunion of femoral shaft and femoral neck, both, can be obtained using a tensioned Schanz pin-based external fixator without the need for any secondary procedure. Such a fixator and cement spacer assembly can thus address the dual purpose of fracture stabilization during infection control as well as the union of the non-union sites.

## Introduction

Concomitant ipsilateral femoral neck and shaft fractures are uncommon injuries with difficult management and a high risk of complications related to the healing of the fracture, and soft tissue injury [1]. The extent of soft tissue injury can often be underestimated in the initial phase and can result in wound complications. Additionally, due to the inherent instability of this fracture pattern, the fixation may fail prior to the achievement of fracture union. Infected nonunion of fracture is another entity that frequently complicates the fracture healing process [2]. The management of infected fracture nonunions has never been simple, and often requires staged management in the form of infection control measures followed by the definitive fixation. In spite of the measures taken to control the infection, future risk of infection can not be ruled out. The infected bone has a poor healing capacity, and the infected segment may need to be excised with further measures for bone transport for the defect created [3]. These procedures are cumbersome and may take several months to regenerate bone. Such infected non-unions should preferably be managed with staged surgeries where the intramedullary and local infection is controlled with local and intramedullary antibiotic cement spacers and temporary stabilization with an external fixator [4]. Additionally, prolonged antibiotic therapy is required according to the culture reports. 

Infected non-union affecting the fracture of femoral shaft and concomitant non-union of the ipsilateral neck of femur fracture poses a challenging situation, and has not been discussed previously in the literature. We report a case of chronic infected nonunion of shaft of femur and concomitant nonunion of ipsilateral femoral neck of eight months evolution subsequently treated with an intramedullary antibiotic cement spacer, extraosseous antibiotic cement beads, and an external fixator stabilizing both the fractures, and that was tensioned in compression on the outer aspect to increase stability. Interestingly, the infection was well controlled with normalization of inflammatory markers, and the union of both fractures was achieved within eight months of the aforestated surgery without the need for any additional procedure. 

## Case presentation

**Figure 1:**
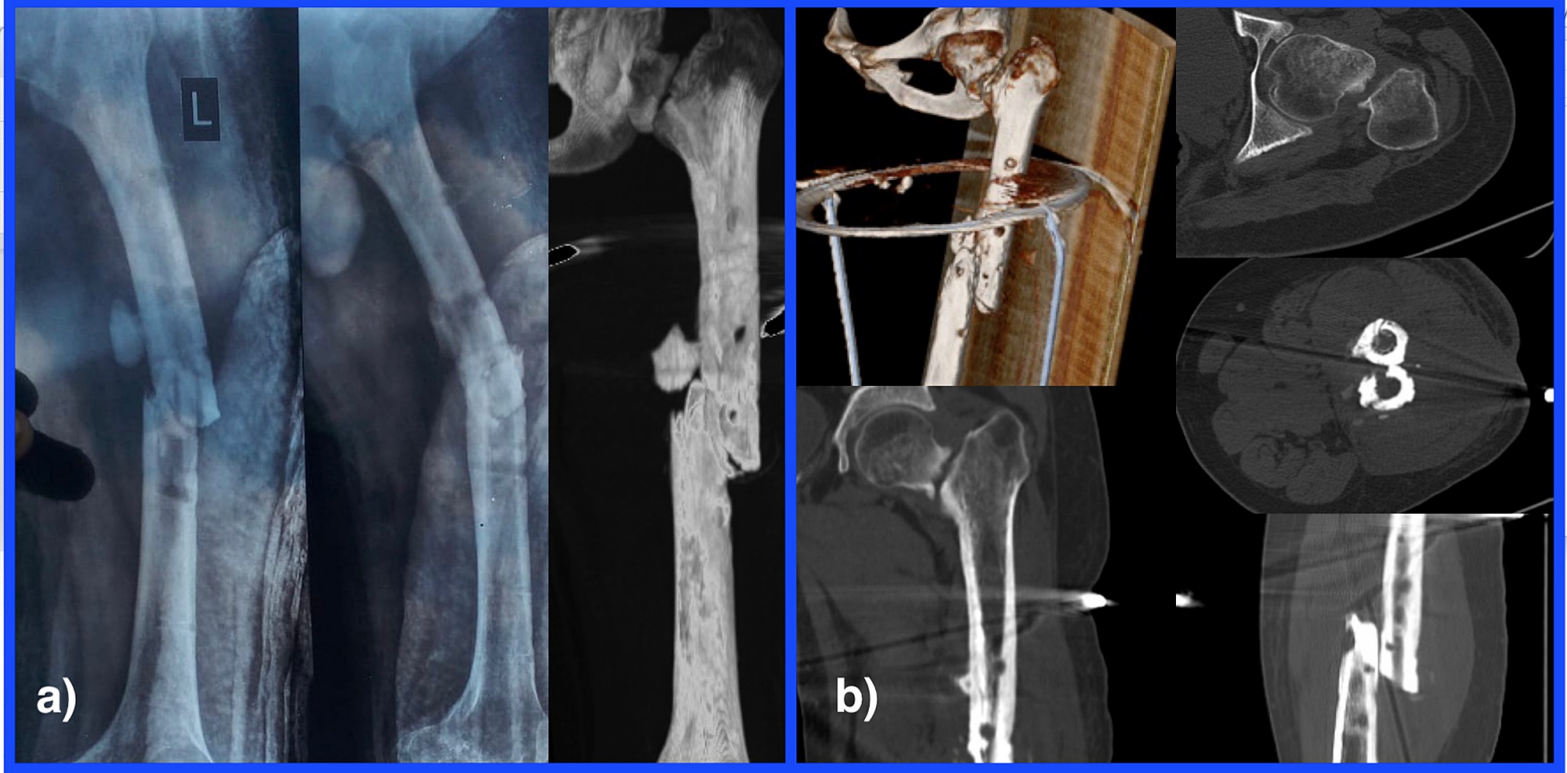
Non-union of the ipsilateral neck of femur and shaft of femur left side. a) Preoperative radiographs reveal non-union of shaft femur fracture without any evidence of healing. The neck of the femur radiograph reveals sclerotic and smooth margins along with the gap at the fracture site. b) the computed tomography 2D and 3D images confirm the findings of atrophic non-union of the shaft of femur and neck of femur.

A 22-year-old male patient, a labour worker by occupation, had presented to our outpatient department with a diagnosis of concomitant nonunion of the shaft and neck of the left femur of eight months duration. On the basis of history and previous records, it was known that the patient had sustained an open grade IIIA fracture of shaft femur and a closed ipsilateral femoral neck fracture of the left side following road traffic eight months prior to the above presentation. The femoral shaft fracture was managed with an external fixator application, and the femoral neck fracture management was delayed due to unhealthy soft tissue conditions around the proximal lateral aspect of the thigh. The treatment of both fractures was further delayed because the patient developed an infection of the open wound of the femoral shaft fracture. To add to the complications, he had developed pin site infection as well. Following that, open debridement of the femoral shaft wound site was performed, and the external fixator was removed at the primary treating institution. Thereafter, the limb was kept splinted on a Thomas splint. Subsequently, the wound and pin sites were healed and the patient was referred to our center, which is a tertiary care center and teaching medical institute, after a total duration of eight months following injury. Clinicoradiological examination confirmed the non-union of the left femoral shaft fracture and ipsilateral femoral neck fracture (Figure 1a). Blood investigations revealed raised serum inflammatory markers (erythrocyte sedimentation rate (ESR)=25 mm/hr and C-reactive protein (CRP)=12 g/L). However, there were no clinical signs of local inflammation around the fracture sites, the healed wound, and the pin sites. An MRI evaluation of the hip joint and the affected thigh was planned but was deferred as the patient was claustrophobic and not comfortable. Instead, an ultrasound evaluation along the femoral shaft and the hip joint was performed, which revealed nil collection or edema. A CT evaluation was performed to evaluate the femoral neck fracture and femoral head to look for any resorption of fracture ends, and any signs of bony abnormality in the femoral head. The hip joint space was normal, sufficient length of the neck in the proximal head fragment was preserved and there were no signs of lucencies, sclerosis, or resorption in the femoral head (Figure 1b). We, therefore, assumed that the femoral head to be viable and salvageable.

**Figure 2:**
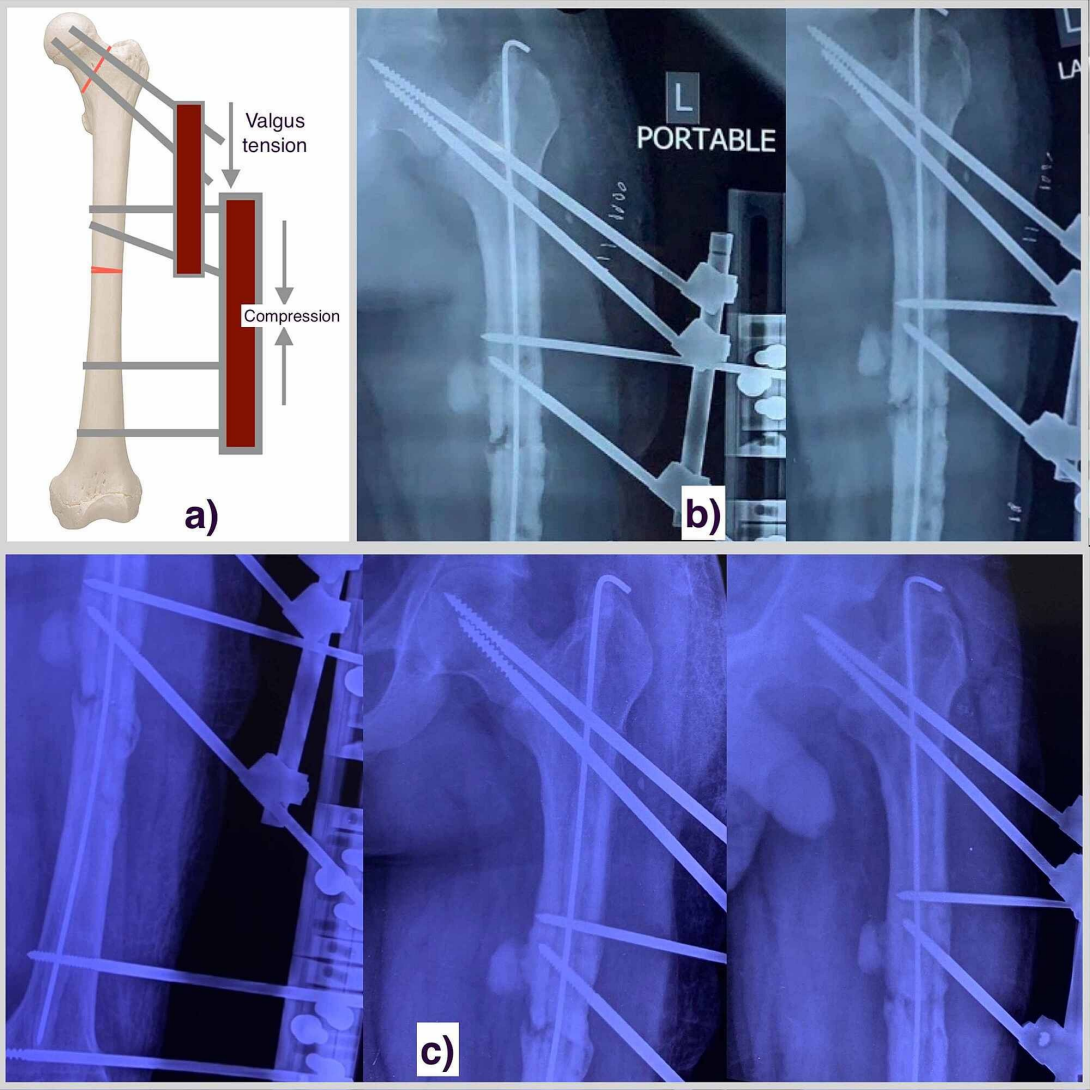
a) A representation image showing the external fixator assembly for the stabilization of the femoral neck as well as femoral shaft non-union. b) The 6 weeks post-operative radiograph ( post cement bead removal) of the left side femoral shaft and hip region suggestive of maintained fracture alignment of both the regions. c) The 3 months post-operative radiograph showing bridging bone formation at the femoral shaft nonunion site, while the union status of femoral neck can't be commented upon.

We reserved our plan for definitive fixation with bone grafting or staged fixation depending upon the exposure of the fracture site. Considering the need for open reduction of both the fracture sites we positioned the patient on a fluoroscopically translucent standard operating table. The femoral shaft fracture was exposed through the previous debridement scar site that was located anterolaterally. Contrary to the clinical evaluation, the medullary cavity on both sides of the fracture was filled with pus with no extraosseous collection. Samples were taken for culture and sensitivity testing. The medullary canal and the surrounding bone were thoroughly lavaged and debrided. The previously paced external fixator pin sites were curetted and were found to be healthy. After thorough femoral shaft debridement, further lavage was performed after reaming of the intramedullary cavity proximally up to the greater trochanter and distally up to the condylar region in order to further clean the canal of any necrotic or infective debris. Thereafter, the anterolateral incision was further extended proximally towards the greater trochanter tip. The canal was entered through the trochanteric tip. We inserted a rush nail based cylindrical antibiotic cement spacer into the medullary canal up to the reaming site in the distal fragment. Antibiotic simplex® cement was used to prepare the cement spacer which consists of colistin and erythromycin. The previous wound culture reports were suggestive of *Klebsiella pneumoniae* that was sensitive to colistin. Along with that, we placed antibiotic cement beads in the surrounding extraosseous space as well. The femoral neck fracture was not exposed considering the risk of contaminating the joint with distal infection. We rather attempted aspiration of the affected hip joint from a sterile zone. Only a minimal amount of clear joint fluid could be aspirated that was also sent for culture sensitivity testing. Following that, the shaft femur fracture was stabilized by an external rail fixator with antibiotic cement coated pins proximally and distally. There was a medial defect at the shaft femur nonunion site, and therefore, the rail fixator was tensioned in compression laterally to prevent varus failure (Figure 2).

**Figure 3:**
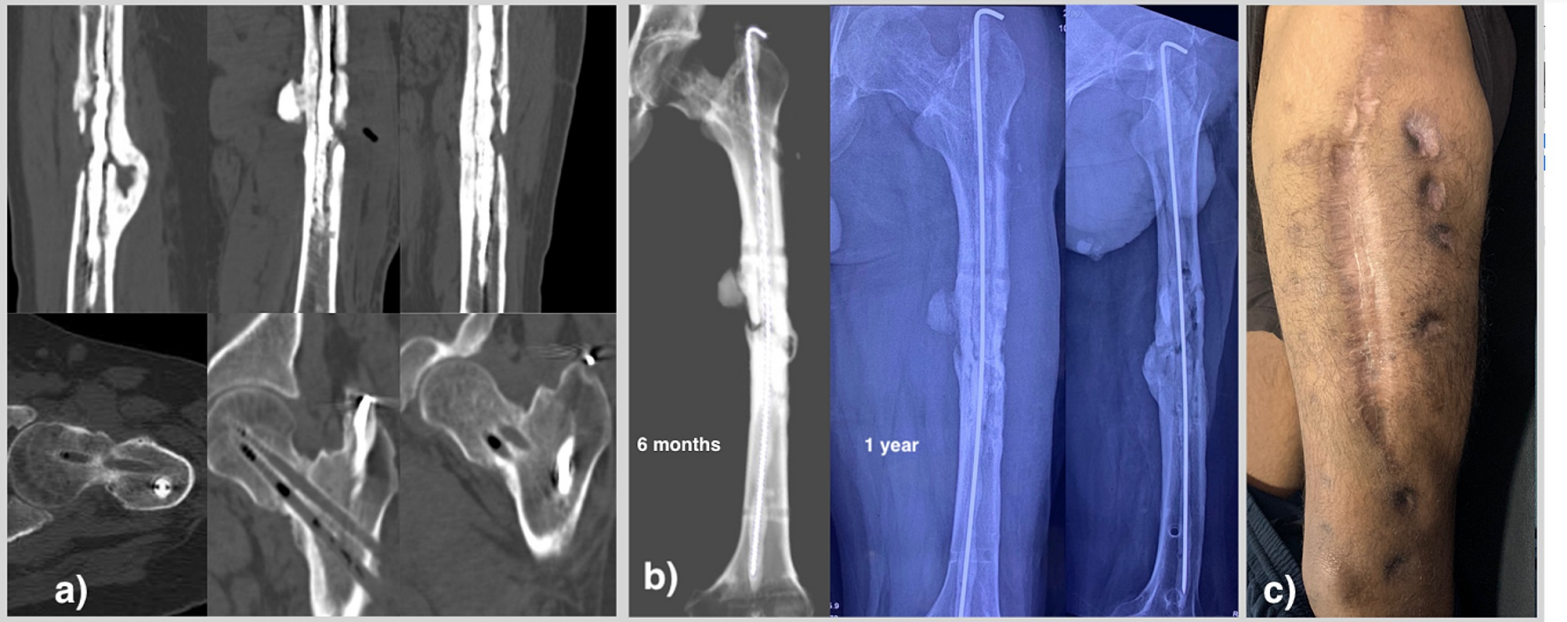
a) The CT images at the 6-month postoperative period suggest at least three cortices united for the femoral shaft and complete union of the femoral neck. b) The 6 month and one-year follow-up radiographs of the same patient showing consolidation of both fracture sites (femoral neck and femoral shaft). c) The clinical picture showing healing of all wounds and pin sites.

The femoral neck fracture was aligned under fluoroscopic guidance and a positive inferomedial cortical edge was induced to prevent varus failure. The femoral neck fracture was also spanned by two antibiotic cement-coated Schanz screws. The lateral ends of those pins were incorporated in the rail external fixator and tensioned in valgus force to prevent varus collapse of the femoral neck. The wound was closed in layers without drain after meticulous hemostasis. Empirical intravenous antibiotic therapy based on previous culture reports was initiated in the immediate postoperative period. Thereafter, according to the culture isolates (coagulase-negative *Staphylococcus aureus* and *K. pneumoniae*), we initiated intravenous antibiotics, tigecycline, levofloxacin, and clindamycin, and continued them for three weeks. The systemic impact of antibiotic therapy was monitored periodically and was uneventful. The patient was discharged on sensitivity-based oral antibiotics for three more weeks. The hip joint aspirate was found to be sterile upon culture testing. The postoperative wound healed without any complications and pin sites remained healthy and were regularly cleaned. The antibiotic beads were removed after six weeks. The patient was mobilized using a walker with a toe touch stance on the affected side. Our plan was to remove the intramedullary cement spacer and the external fixator at three months duration anticipating the inflammatory parameters to be normalized by that time. However, the plan to remove the intramedullary antibiotic cement spacer was deferred after the evaluation of the follow-up radiographs (Figure 2c). The radiographs revealed bridging bone formation from the bone adjacent to the fracture margins. Also, the gap at the femoral neck fracture site appeared reduced. While we did not anticipate the healing of the femoral neck fracture, the callus formation at the shaft fracture was a good sign for the possibility of fracture union. The blood counts and inflammatory parameters were all normal at this stage. The patient was further followed for another three months duration, and there was sequential progress in the bridging bone formation around the shaft fracture site and no gap was appreciable at the femoral neck fracture site throughout the follow-up (Figure 3).

Interestingly, the medial defect at the shaft fracture also had the signs of bridging bone formation. However, the patient complained of pin loosening. The rail fixator was thus removed. Post fixator removal, the patient had no symptoms at hip and the shaft fracture site. Pin sites were free of any discharge. Since the radiographs were insufficient to precisely evaluate the status of femoral neck fracture and only biplanar healing of the shaft fracture could be evaluated, a CT scan was ordered after the fixator removal. Surprisingly, the CT scan revealed a complete union and consolidation of the femoral neck fracture nonunion, and more than three-fourths of cortical bridging of the femoral shaft fracture. Based on this finding, the patient was allowed a partial weight-bearing for a month and full weight-bearing from the next. The most recent radiographs revealed complete further consolidation. Currently, after one year of the last surgery, the patient is not on any antibiotic therapy and the levels of the inflammatory marker are within normal limits. The patient had some stiffness of the knee that has been recovering with regular exercises. The patient had been advised for the removal of the intramedullary cement spacer. However, the patient has opted for a later date for the removal of the intramedullary cement spacer. The hip movements are completely normal and painless. The patient is able to walk without any limitation, and also able to perform labor activities.

## Discussion

Infected nonunions pose a challenging scenario, often delaying the definitive management of the fracture. The metal fixation devices carry a future risk of reactivation of infection due to biofilm production [5]. Classically, the long bone infected nonunions have been treated using multi-staged as well as single stages procedures [6]. Thorough debridement, local infection control, and rigid stabilization are the main principles in achieving fracture union in such cases. The non-staged procedures can be performed with aggressive infection control and fracture fixation in a single setting. However, more evidence is needed to establish the effectiveness of non-staged procedures. The staged procedures with the first procedure for controlling the local infection, and second for definitive fixation are well-established techniques which are time tested in the management of infected non-union [7,8]. The placement of intramedullary antibiotic cement rods followed by exchange nailing for definitive fixation is an attractive alternative for the management of intramedullary infection [9]. Similarly, antibiotic cement-impregnated nails can also be used for intramedullary infection control. However, at times, it can be challenging to remove the antibiotic cement-impregnated nails from the medullary canal. The rod-based cement spacers consume less space in the medullary canal with a comparable amount of cement volume. The excision of the infected segment of bone, especially with defects, can also be managed using induced membrane technique, and by bone-transport using Ilizarov or rail rod fixation. However, in cases with intramedullary infection, that can potentially be associated with multiple pin sites infection following external fixator application, the aforestated techniques may not be able to eradicate infection. A thorough debridement and intramedullary infection control will be required in such cases.

Concomitant fractures of the femoral neck and ipsilateral femoral shaft belong to high energy trauma and complication rates are high. There is a higher risk of nonunion of femoral neck fracture [10]. Also, the non-unions of the femoral shaft in these injuries take a much longer time to heal. Multiple surgeries are often needed and patient morbidity is high. An infected nonunion in such injury and with none of the fractures united poses a more challenging scenario and outcomes can be difficult to predict considering the scarcity of evidence in dealing with such situations.

Due to previous inexperience and lack of evidence from literature in dealing with the concomitant nonunion of the femoral neck and shaft fractures with intramedullary infection, we utilized the basic principles in the management of such injuries. 

The patient was positioned supine on a standard operating table. We had no previous experience of performing open reduction of these two fractures on a traction table. Additionally, the traction table would have prevented the manipulation of the proximal and distal segments for length restoration as well as the open reduction of the femoral neck fracture.

For the intramedullary infection, we planned for an intramedullary antibiotic cement spacer for the high local concentration of antibiotics, and for local infection control of the surrounding soft tissue, we curetted the pin sites and placed antibiotic cement beads around the femoral shaft. To maintain the integrity of the intramedullary cement spacer and prevent the risk of breakage, the mantle was prepared over a rush nail/rod. We avoided using thicker alternatives like K nail so as to avoid difficulty in second surgery where the cemented nail may need to be exchanged with an interlocking nail. Additionally, we wanted to stabilize the fracture with maximal bony contact at the fracture fragments, so that minor movements of the limb and non-weight-bearing mobilization could be started without stress transfer around the fracture site. Therefore, a rail fixator frame was applied with maximum possible compression at the fracture site. A bony contact and low strain environment with stable support can help in bone formation. The interfragmentary strain theory explains what tissues can form depending upon the gap and tension at the fracture site [11]. The construct that we used was a stiff one with the aim to provide a low strain environment that favours bone formation. This is also helpful in the event of secondary definitive stabilization. The reaming was performed to remove any intramedullary adhesions that could be a potential source of infection. Additionally, reaming improves the periosteal blood flow and thus can be helpful in the healing process as well. While the above steps seemed satisfactory for the nonunion of the femoral shaft, for addressing neck femur fracture, additional stabilization was required. The hip joint ultrasound didn't reveal any collection and the intraoperative aspiration was also negative. Therefore, we opted to not open the hip joint to avoid the transfer of any infected focus. The CT findings did not show any abnormality in the joint or femoral head, except for the fracture margin sclerosis. The femoral neck length was sufficient to be salvaged, and therefore we kept the option of definitive osteosynthesis at a later stage when the femur shaft infection subsides. Since there was a clear fracture nonunion space at the femoral neck non-union site, we required minimal traction and rotational control only to realign the fracture. At that stage we didn't plan for an anatomical reduction as that would have required open reduction and could potentially have contaminated the neck fracture as well.

**Figure 4:**
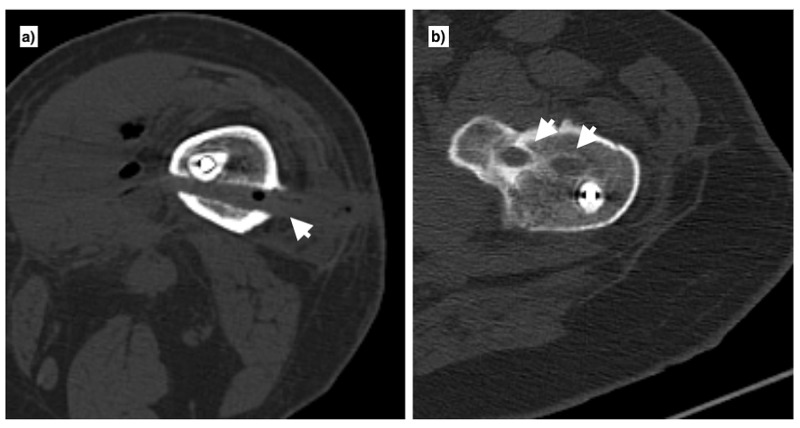
a) The cross section of femoral shaft at isthmus showing space for the schanz pin that was placed posterior to the antibiotic cement rod (single arrowhead). b) The femoral neck schanz pins were placed slightly anterior to the mid lateral aspect of proximal femur to avoid hindrance with the cemented rod (double arrowheads).

However, we wanted the femoral neck fracture to be well stabilized and not fail in varus collapse. A lack of stabilization of the femoral neck fracture could have resulted in further resorption of the neck and secondary changes in the hip joint as well. We, therefore, placed two antibiotic cement-coated Schanz screws in the superoinferior plane to provide rotation stability to the fracture after obtaining a positive medial cortical hinge to prevent varus collapse, and incorporating the outer ends of the Schanz screws in the rail rod frame. The Schanz screws were tensioned in valgus and locked to the rail frame. The shaft and neck fractures were found to be stably positioning with the hip movements. The placement of pins through the femoral neck from the proximal lateral femoral cortex is not a straightforward technique. The technique utilizes the basic knowledge of the anatomy of the proximal femur. The greater trochanteric tip which was the entry point for the cemented rush rod actually lies posterior to the central axis of the femoral shaft. Also, the proximal femur is broad in both frontal and sagittal planes. The femoral neck is anteverted in relation to the femoral shaft. Therefore, the drilling for the pins need to be done from the anterior half of the lateral cortex, and directed anteromedially to match the anteversion of the femur (Figure 4a). Fluoroscopic assistance is beneficial in directing the pins. Also, as the pins of the femoral neck converge downwards laterally, they reduce the space for the placement of the proximal femoral shaft pins. This problem can be overcome by using a combination of obliquely directed pins and transverse pins of longer lengths sufficient enough to place the rail rod frame away from the femoral neck pins. The femoral neck pins can be connected and tensioned to the shaft pins using a separated clamp and rod assembly. As far as the femoral medullary canal is concerned, the assembly takes the advantage of the wider anteroposterior extent of the medullary canal even at the isthmus compared to the mediolateral extent. The cemented rush nail was kept to an order of 8 mm in total and the prior reaming with 13 mm reamer provided sufficient space for Schanz pin placement anterior or posterior to the rod-based cement spacer. Depending upon the orientation of the rail frame, the entry point for the drill bit can be made anterior or posterior to the cemented rod with guidance from the lateral fluoroscopic view. Figure 4 shows the space available for the placement of the pin in an axial cut of the femoral medullary canal at its isthmus.

The patient was mobilized with toe touch walker-assisted mobilization to put a low amount of physiological stresses around the fracture sites. We feel that stability at the fracture sites, bony contact at the femoral shaft fracture, and toe touch mobilization leading to physiological stresses around the fracture sites, and the construct stability contributed to the union of the two fractures.

Concerning the femoral neck fractures nonunion, the literature suggests that they need some realignment osteotomy and bone grafting for successful outcomings, provided that the femoral head is viable. External fixator has been described for intertrochanteric femoral fractures in situations where internal fixation can not be performed. However, nonunions are rare in this region owing to the high vascularity and cancellous bone. Contrary to this the intracapsular fractures of the femoral neck have precarious vascularity and are at risk of nonunion for the same reason. However, the nonunion of the femoral neck can be brought to the union without exposing the fracture by realignment osteotomies like valgus intertrochanteric osteotomy. The osteotomy brings the femur neck fracture to a mechanical advantageous orientation that helps in the union by increasing the compressive forces across the fracture site. The important role of mechanical stresses across the femoral neck fractures understood from the fact that the fixation devices for these fractures are still evolving with more stress on stability. The Schanz pins placed in the femoral neck in the current case were not independent of the femoral shaft fixator frame. Otherwise, their placement would have been similar to the placement of two cancellous screws. Here we added two stabilizing forces across these pins. First, we connected the pins with the rail rod frame via an additional clamp. Secondly, we added valgus tension on these pins that prevented varus collapse. This biomechanically favourable environment probably helped in achieving union at the femoral neck nonunion site without the need for any additional augmentation procedure.

To the best of our knowledge, management of chronically infected non-union of concomitant ipsilateral femoral shaft and neck fractures has not been reported and described in the past. The current report and management technique, on one hand, can be helpful in controlling intramedullary infection, also provides a simple treatment option for achieving union at both the fracture sites. The case presentation also points towards the effectiveness of rigid stabilization in achieving union in chronic nonunion of the femoral neck which itself is termed as a problem fracture owing to the peculiarities in the outcomes and ever-evolving modalities for its fixation.

## Conclusions

Intramedullary antibiotic cement spacers are among the established treatment modalities to control intramedullary infected non-unions of long bones. This treatment modality combined with a tensioned external fixator using antibiotic cement coated Schanz screws and rail rod frame for spanning both the fractures is a simple and attractive alternative to control the infection and can potentially negate the need for further surgical intervention for the definitive fixation for the infected non-union of ipsilateral femoral neck and shaft fractures.
